# Role of healthcare workers in early epidemic spread of Ebola: policy implications of prophylactic compared to reactive vaccination policy in outbreak prevention and control

**DOI:** 10.1186/s12916-015-0477-2

**Published:** 2015-10-19

**Authors:** Cordelia E. M. Coltart, Anne M. Johnson, Christopher J. M. Whitty

**Affiliations:** Research Department of Infection and Population Health, Institute of Epidemiology, UCL, London, UK; Clinical Research Department, London School of Hygiene & Tropical Medicine, London, UK

**Keywords:** Ebola, Healthcare worker, Prevention, Vaccination

## Abstract

Ebola causes severe illness in humans and has epidemic potential. How to deploy vaccines most effectively is a central policy question since different strategies have implications for ideal vaccine profile. More than one vaccine may be needed. A vaccine optimised for prophylactic vaccination in high-risk areas but when the virus is not actively circulating should be safe, well tolerated, and provide long-lasting protection; a two- or three-dose strategy would be realistic. Conversely, a reactive vaccine deployed in an outbreak context for ring-vaccination strategies should have rapid onset of protection with one dose, but longevity of protection is less important.

In initial cases, before an outbreak is recognised, healthcare workers (HCWs) are at particular risk of acquiring and transmitting infection, thus potentially augmenting early epidemics. We hypothesise that many early outbreak cases could be averted, or epidemics aborted, by prophylactic vaccination of HCWs. This paper explores the potential impact of prophylactic versus reactive vaccination strategies of HCWs in preventing early epidemic transmissions. To do this, we use the limited data available from Ebola epidemics (current and historic) to reconstruct transmission trees and illustrate the theoretical impact of these vaccination strategies. Our data suggest a substantial potential benefit of prophylactic versus reactive vaccination of HCWs in preventing early transmissions. We estimate that prophylactic vaccination with a coverage >99 % and theoretical 100 % efficacy could avert nearly two-thirds of cases studied; 75 % coverage would still confer clear benefit (40 % cases averted), but reactive vaccination would be of less value in the early epidemic.

A prophylactic vaccination campaign for front-line HCWs is not a trivial undertaking; whether to prioritise long-lasting vaccines and provide prophylaxis to HCWs is a live policy question. Prophylactic vaccination is likely to have a greater impact on the mitigation of future epidemics than reactive strategies and, in some cases, might prevent them. However, in a confirmed outbreak, reactive vaccination would be an essential humanitarian priority.

The value of HCW Ebola vaccination is often only seen in terms of personal protection of the HCW workforce. A prophylactic vaccination strategy is likely to bring substantial additional benefit by preventing early transmission and might abort some epidemics. This has implications both for policy and for the optimum product profile for vaccines currently in development.

**Electronic supplementary material:**

The online version of this article (doi:10.1186/s12916-015-0477-2) contains supplementary material, which is available to authorized users.

## Background and policy question

Ebola causes severe illness in humans, with a high case-fatality rate [1–3] and epidemic potential, illustrated by the recent West African outbreak. There have been 29 outbreaks of Ebola reported since it was first identified in 1976 (predominantly from *Zaire* (15) and *Sudan* (7) strains, which account for the vast majority of human disease; Table 1) [1]. The outbreak in West Africa is larger than all other outbreaks combined, with 28,183 reported cases and 11,306 deaths by 6 September 2015 [4]. The impact on individuals, healthcare, societies, and economies has been profound.

**Table 1:** Chronology of Ebola *Zaire* strain outbreaks [1, 4]

Country	Outbreak location	Year	Number of cases	Number of deaths
Democratic Republic of Congo (DRC)	Yambuku	1976	318	280
DRC	Tandala	1997	1	1
Gabon	Mekouka	1994	52	31
DRC	Kikwit	1995	315	254
Gabon	Mayibout	1996	37	21
Gabon	Booue	1996	60	45
Gabon	Mekambo	2001–02	65	53
Republic of the Congo (RC)	Mbomo Kelle	2001–02	57	43
RC	Kelle	2003	143	128
RC	Mbandza Mbomo	2003	35	29
RC	Etoumbi	2005	12	10
DRC	Luebo	2007	264	187
DRC	Mweka and Luebo	2008–09	32	15
DRC	Jeera	2014	66	49
Multiple countries [4]	West Africa	2014–15	28,183	11,306

Preventing and mitigating future epidemics is a public health priority and an effective vaccine would be an important tool to respond to future outbreaks. Several vaccines are currently being trialled or are in development. How to deploy any vaccine most effectively is a central policy question and each strategy has different implications for ideal vaccine characteristics and profiles. In this paper, we set out to explore the differential impact and policy implications of two vaccination strategies of healthcare workers (HCWs) and associated workers: prophylactic (pre-epidemic) vaccination compared to reactive vaccination once an epidemic has started. Prophylactic vaccination would not be a trivial undertaking, so should only be considered if there are potentially significant benefits.

HCWs^1^ [5], have a high incidence of severe disease and deaths from Ebola during epidemics [1], accounting for up to 25 % of reported cases across historic (smaller) epidemics [6, 7] and a case attack rate of up to 31 % for physicians reported in a single epidemic [8]. In the recent West African outbreak, prior to the provision of good equipment and precise attention to infection control procedures, the relative risk for acquiring Ebola was around 100 times higher for HCWs compared to the general population [9], although this risk can be substantially reduced with the correct use of barrier precautions and personal protective equipment [10, 11]. However, by the time Ebola is suspected, diagnosed, and precautions implemented, many exposures and infections have already occurred as the incubation period is relatively short (2–21 days, average 11 days) [12] and transmissibility is high from severely ill patients in healthcare settings. Therefore, in these early stages of an epidemic, before the outbreak has been recognised, HCWs not using personal protective equipment are at substantial risk and often constitute a high proportion of early cases. There is an obvious humanitarian case to vaccinate HCWs in potentially affected countries during an outbreak for their own protection and, if an effective and relatively safe vaccine exists, there is little doubt it will be used for this group reactively, so this is not a major uncertainty for policy. It is less clear whether prophylactic vaccination between epidemics has a role. A vaccine optimised for prophylactic vaccination would have significantly different characteristics to a reactive vaccine: in particular a prophylactic vaccine would need to have longevity of protection and few side effects, whilst a reactive vaccine would have to prioritise speed of onset of protection.

In many epidemics, HCWs have been the transmission link to the general population, acting as ‘super-spreaders’ in early epidemics. The importance of this HCW transmission pathway appears to predominate in early epidemic transmission chains and decreases as the epidemic evolves [13, 14], probably due to effective infection control measures in healthcare settings once an epidemic is recognised. There are a number of potential reasons that HCWs are both more likely to acquire and transmit Ebola. For example, the occupational risks of acquiring the disease are high, given close contact with patients who are highly infectious, but not known to be infectious at the time. Further, once acquired, there is an increased risk of spreading infection through societal and cultural factors including that HCWs touch a wide variety of strangers, travel more widely, and may be significant public figures who have large traditional burials. Therefore, as they are a high-risk and relatively easily identified group, it is sensible to consider whether a pre-epidemic specific vaccination strategy for HCWs could have an epidemic-modifying role in addition to a humanitarian one.

Partly as a result of Ebola being recognised as a potential bioterrorism agent [15, 16], vaccine development work had begun prior to the recent outbreak. Potential vaccines exist for both the *Zaire* and *Sudan* Ebola virus strains. Recent events in West Africa increased the urgency for an Ebola vaccine and fast-track clinical trials were undertaken [17–19]. Initial data from early trials, recent publication of interim analyses, and non-human primate studies suggest the vaccines are safe, efficacious, and have the potential to be effective at a population level [18, 20, 21]. However, additional evidence from final analyses and the completion of Phase II/III human trials will still be required. Due to the rapidly falling case numbers, efficacy data will probably be limited and vaccine efficacy largely inferred from a non-outbreak setting and immunological correlates of protection.

Whilst the ideal Ebola vaccine would be safe, well tolerated, rapidly effective, and have long duration of high levels of protection, in reality, there may well be a trade-off between different characteristics (e.g. tolerability versus speed of onset versus duration of protection) and more than one vaccine may be needed. At least four possible vaccination strategies are under consideration, which have different ideal vaccine profiles:

1. Ring vaccination of the contacts of cases. This needs to be rapidly effective; duration of protection would be less important. Initial data already supports this approach [20].
2. Mass vaccination of the general population during an outbreak. Here safety and ease of use (e.g. single dose) would dominate as essential characteristics.
3. Reactive vaccination of HCWs caring for patients during an epidemic. The speed of onset of protection and efficacy would be essential given the high risk of transmission; duration of protection beyond a few months and a minor side effect profile would be less important given the high risks associated with infection.
4. Vaccination of HCWs in countries where Ebola is potentially endemic prior to an outbreak as a prophylactic measure. Unlike other vaccination strategies, duration of protection would be central and safety and tolerability would be much more important as it is likely that most vaccinated HCWs would never work in an outbreak setting (rare events) and, therefore, not encounter the virus. Speed of onset of protection would be less important and a two- or three-dose vaccine strategy would be reasonable. Therefore, this strategy could be considered as an outlier in terms of vaccine profile.

There is no question that, if an effective vaccine is developed, it will be used reactively to vaccinate HCWs in an epidemic. However, we hypothesised that the magnitude of outbreaks could be significantly reduced, or even aborted at an early stage, with prophylactic pre-epidemic protection of HCWs. This has to be predicated on a long-lasting, well-tolerated vaccine being available. Given the current speed of development of vaccines and policy, the aim of this paper is a rapid analysis of known and provisional Ebola transmission tree data to assess what could have happened if prophylactic vaccination (4) versus reactive vaccination (3) strategies for HCWs had been deployed and the impact this would have had in preventing early epidemic transmissions and epidemic progression. To inform the debate, descriptive hypothesis generation and illustrative testing of different vaccination strategies for HCWs were undertaken to see if a long-duration vaccine for prophylactic use is sufficiently attractive to be worth prioritising due to its impact on transmission of early epidemics, as compared to reactive vaccination.

## Methods

### Data providing illustrative examples

**Fig. 1:**
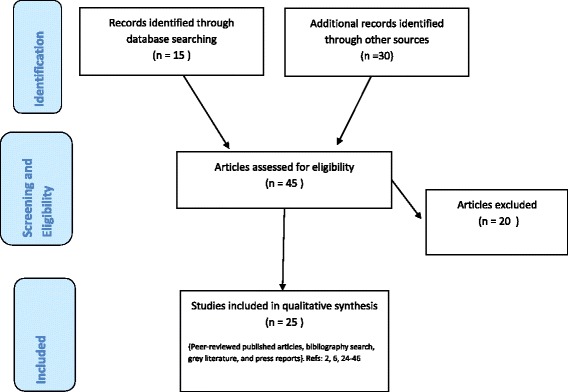
PRISMA Flow Diagram

To examine the hypothesis that prophylactic vaccination could have a significant epidemic-modifying impact on early epidemic transmission, we used data available from the current and historic Ebola epidemics. The methods and search strategy for the review are detailed in Table 2 and Fig. 1. From the articles identified [2, 6, 22–44] we reconstructed the initial epidemic transmission trees for all available previous outbreaks of Ebola *Zaire* virus, initially using published and grey literature and, where these were not available, press reports (see Additional file 1). The transmission trees detail the number of cases as well as who transmitted the virus to whom, and HCW status where known. Therefore, it is possible to estimate the number of cases that resulted from HCW transmission. These transmission trees were then used to assess the proportion of the early known Ebola epidemic cases that would have been averted under various vaccination strategies if HCWs had been protected and had not contributed to transmission chains.

**Table 2:** Methods and search strategy for review and transmission tree reconstruction

Methods	
Search strategy	A compound search strategy was developed to identify all relevant open-source articles regardless of publication status. The initial search was undertaken via PubMed using the search terms outlined below. Further information was obtained by reviewing article bibliographies for relevant citations and a Google search to find open-source published articles, press articles, and other grey literature including outbreak updates, WHO roadmaps, WHO situation reports, and Morbidity and Mortality Weekly Reports from the Centres for Disease Control.
	The purpose of the review is to outline potential policy implications for vaccine development, as opposed to defining detailed epidemic trees.
Search terms used	Key words: Ebola, Ebola haemorrhagic fever, Ebolavirus, Ebola virus disease, transmission, transmission trees, epidemic, epidemic trees.
	(("hemorrhagic fever, Ebola"[MeSH Terms] OR ("hemorrhagic"[All Fields] AND "fever"[All Fields] AND "Ebola"[All Fields]) OR "Ebola hemorrhagic fever"[All Fields] OR "Ebola"[All Fields] OR "Ebolavirus"[MeSH Terms] OR "Ebolavirus"[All Fields]) OR ("hemorrhagic fever, Ebola"[MeSH Terms] OR ("hemorrhagic"[All Fields] AND "fever"[All Fields] AND "Ebola"[All Fields]) OR "Ebola hemorrhagic fever"[All Fields] OR ("Ebola"[All Fields] AND "virus"[All Fields] AND "disease"[All Fields]) OR "Ebola virus disease"[All Fields])) AND ((("transmission"[Subheading] OR "transmission"[All Fields]) AND ("trees"[MeSH Terms] OR "trees"[All Fields] OR "tree"[All Fields])) OR (("epidemics"[MeSH Terms] OR "epidemics"[All Fields] OR "epidemic"[All Fields]) AND ("trees"[MeSH Terms] OR "trees"[All Fields] OR "tree"[All Fields])))
Inclusion criteria	Studies and articles were eligible for inclusion if they reported human-to-human transmission chains and initial transmission chains from the primary or index case within each outbreak or country in multi-location epidemics. Details of occupational exposure of individuals in the transmission trees were also included where possible. There were no restrictions with regards to date of publication. The aim was to identify as many early epidemic trees as possible, and to include all which could be reliably identified as including HCW status to minimise bias.
Data extraction	Data extraction was first undertaken using peer-reviewed literature. These data were supplemented by grey literature and press articles to add further information and fill in the gaps for the epidemic tree construction.
	Approximate numbers have been used where precise numbers were not available, but in all cases conservative assumptions have been made to avoid overestimation of any effects identified.
Data analysis	Initial analyses of the different vaccination strategies were undertaken in Microsoft Excel (2010). Using the initial transmission trees constructed, the number of cases that developed the disease and the number of cases averted were calculated for each vaccination strategy. All results are given as a percentage of averted cases by the total number of cases. The results are given per vaccination strategy and epidemic location.

Of the 15 *Zaire* strain Ebola virus outbreaks, initial epidemic transmission trees of varying detail were possible to reconstruct for eight outbreaks. Only two studies outlined transmission trees directly (Guinea and Nigeria epidemics of the West Africa outbreak [22, 23]), while all others were constructed using multiple data sources and linking this information to reconstruct a ‘best approximation’ tree. For many of the historical outbreaks there was no detailed person-to-person transmission information to enable construction of transmission trees, only hypothetical transmission scenarios which were subsequently excluded. Therefore, our main testing of the hypothesis is based on three outbreaks: the recent outbreak in West Africa (including all nine countries in which cases have occurred, each considered as a separate transmission tree based on an imported primary case); and two historical outbreaks (one with two separate transmission trees from two geographically distinct outbreaks, but originating from the same source)(all transmission trees used in the illustrative analysis are included in Additional file 1). The historical outbreaks were selected according to those outbreaks with transmission trees detailed enough to allow the proposed analysis (Yambuku 1976, Kikwit 1995, and Mosango 1995). Transmission trees from later in the outbreak are available but have not been included as this study focuses on early transmission. It is clear that once an epidemic is established, spread from HCWs (as opposed to spread to HCWs) becomes a minor route of transmission with transmission by other means, such as burials and community transmission, predominating [13]. Therefore, vaccination of HCWs in this later epidemic setting, whether prophylactic or reactive, would have a minimal impact on the epidemic.

Our analysis investigates four different vaccination strategies of HCWs to illustrate the impact of a prophylactic versus a solely reactive HCW vaccination strategy:

### Strategy 1

Prospective prophylactic vaccination of all front-line HCWs in high-priority areas prior to an epidemic and those likely to be deployed to epidemic areas, assuming >99 % vaccination coverage and vaccination efficacy. In this idealised scenario, all cases of HCW infection were prevented, as were the cases arising from transmission by HCWs.

### Strategy 2

Prophylactic vaccination as above with only 75 % coverage (more realistic). As not all HCWs are vaccinated in this scenario the anticipated outcome of the vaccination strategy varied depending on which HCWs were not vaccinated. In all transmission trees the HCWs chosen for vaccination were those associated with the fewest linked transmission cases, except in the situation in which a HCW was not documented to have transmitted the disease. For example, where 75 % vaccine coverage is analysed, the 25 % of HCWs (i.e. one in four) in the transmission tree who were not vaccinated were deliberately selected to minimize the number of cases averted. This method minimises the possibility of overestimating the efficacy of a vaccination strategy by giving the most conservative estimates (‘worst case scenario’ with the highest onward transmission occurring).

### Strategy 3

Reactive rapid vaccination of all front-line HCWs once an epidemic has been identified. We assume an (optimistic) lag-time of 42-days from case presentation to immune protection from vaccine response. This is based on a 28-day logistical window from epidemic identification to initiation of a vaccination campaign, followed by a 14-day lag-time for immune protection following vaccination. This is inferred from the recent interim report of the Guinea ring vaccination cluster-randomised trial of an rVSV-vectored vaccine expressing Ebola surface glycoprotein in which there were no cases reported 10 days after vaccination, implying immune protection [20]. To calculate the cases averted by reactive vaccination, the dates of infection were analysed. Any HCW presenting more than 42 days after the date of the index case was considered to be potentially vaccinated and protected, thus preventing infection and any subsequent transmission.

### Strategy 4

Extension of strategy 1 (prophylactic vaccination) to include traditional healers as well as front-line HCWs. Therefore, all cases of HCWs, traditional healers, and subsequent ongoing transmission from both these groups would be prevented.

## Results

**Fig. 2:**
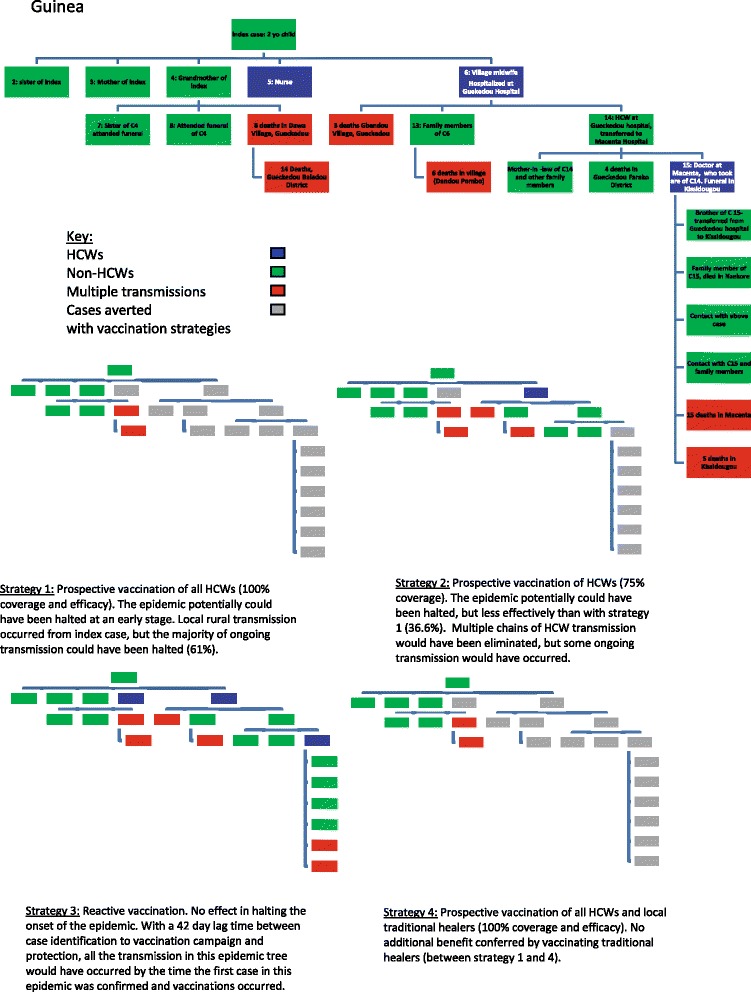
An example of epidemic transmission trees and the impact of four different vaccination strategies on the transmission chains. Guinea 2014 outbreak [22]

**Fig. 3:**
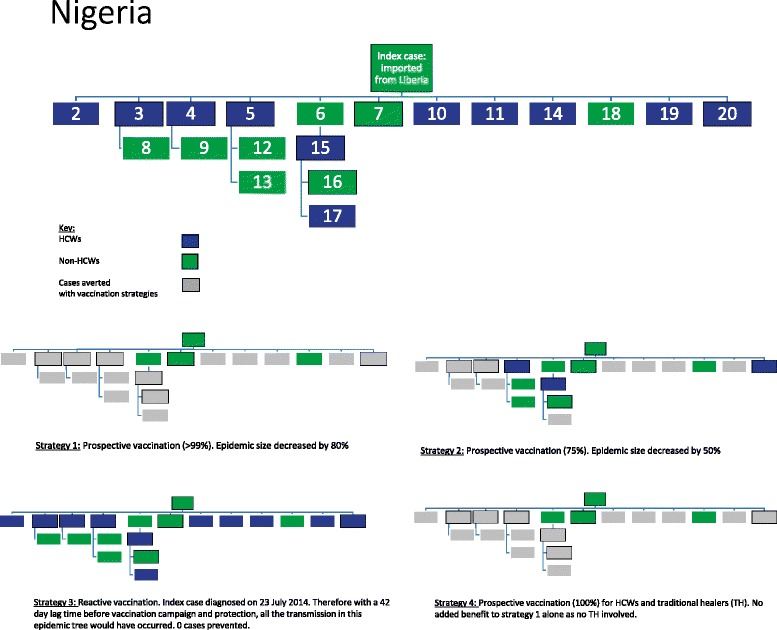
An example of epidemic transmission trees and the impact of four different vaccination strategies on the transmission chains. Nigeria 2014 epidemic [23]

Figures 2 and 3 shows visual examples of epidemic trees and the analysis undertaken, and illustrates the potential impact of each vaccination strategy on transmission. Table 3 shows the putative impact of the first three vaccination strategies outlined by epidemic location. It details the cases averted had HCWs been vaccinated and therefore protected:

**Table 3:** Indicative proportion of early outbreak prevented by implementing different vaccination strategies: prospective versus reactive vaccination of healthcare workers

					Percentage of initial outbreak prevented by vaccination strategy		
Epidemic	Country	Total number of cases [4]	Total number of deaths [4]	Cases included in epidemic tree	Strategy 1: Vaccinate prophylactically (100 % coverage)	Strategy 2: Vaccinate prophylactically approx. 75 % of HCWs	Strategy 3: Vaccinate reactively (lag-time 42 days)
2014 West Africa	Guinea [22]	3,792	2,530	71	61 % (43/71)	36.6 % (26/71)	0
	Liberia [24–28]	10,672	4,808	9	67 % (6/9)	11 % (1/9)	0
	Sierra Leone [29–31]	13,683	3,953	NR	NR	NR	0
	Nigeria [23]	20	8	20	80 % (16/20)	50 %(10/20)	0
	Mali [32–34]	8	6	8	38 % (3/8)	13 % (1/8)	0
	USA [35]	4	1	4	75 % (3/4)	50 % (2/4)	0
	UK [36] and Spain [37]	2	0	2	100 % (2/2)	50 % (1/2)	0
	Senegal [38]	1	0	1	0	0	0
	Overall	28,183	11,306	115	63.5 % (73/115)	35.7 % (41/115)	
	(95 % confidence interval)				(0.54–0.72)	(0.27–0.45)	
Historic outbreaks	Kikwit [6, 39–43]	315	250	9	100 % (9/9)	NR	NR
	Mosango [44]	23	18	23	100 % (23/23)	74 % (17/23)	NR
	Yambuku [2]	318	280	45	44 % (20/45)	NR	NR
Total				192	65.1 % (125/192)	42.0 % (58/138)	0.0 % (0/609)
(95 % confidence interval)					(0.58–0.72)	(0.34–0.51)	

Cases numbers accurate as of 06/09/2015

NR, Not reported

### Strategy 1

Approximately two-thirds (65 %, 73/115) of early epidemic cases across three different epidemics (and 12 outbreak locations) would have been averted with prophylactic vaccination of HCWs and a vaccination coverage >99 %. Across all epidemics, this strategy decreased early epidemic transmission by between 38–100 %. The Sierra Leone data [29–31] suggest that at least 25 % (125/506) of early transmissions would have been averted with this strategy, but as this is based on gross approximate numbers with which we were unable to construct a transmission tree, these figures have not been included in our analysis.

### Strategy 2

We assume prophylactic vaccination of approximately 75 % of HCWs. This strategy would have averted 42 % (58/138) of epidemic cases. Across all epidemics, the percentage of cases averted ranged from 11–74 %. There will be a drop-off point in which vaccine coverage falls below critical levels, but further work would be needed to model this. This data is based on only two outbreaks (West Africa and Mosango) [22–38, 44] as the other historical outbreaks did not provide enough detailed transmission data to determine exact transmission chains with relation to HCW status.

### Strategy 3

A reactive vaccination strategy was assessed. This strategy was unable to prevent any early cases (0 %, 0/609) and was, therefore, ineffective at mitigating epidemics based on this study data. As only initial transmission trees were used, most of the data does not extend past the third wave of infections and does not detail transmission events after 42 days to fully assess the impact of this strategy on later epidemic transmission.

### Strategy 4

When the vaccination strategy included traditional healers, the effect was context-dependent. It had a potentially large effect (63–100 %) in two regions of the current West Africa epidemic, Sierra Leone and Mali [29–34], but did not appear to have an effect in the majority of locations. Based on this limited information, it is difficult to draw conclusions.

These findings suggest that prophylactic vaccination of HCWs might have led to a significant reduction, or even avoidance, of epidemics by preventing the early epidemic transmissions and the subsequent snowballing cascade which resulted in the exponential growth of cases. A reactive vaccination strategy would have had little impact on the first few of waves of the initial epidemic, and by the time a reactive strategy was effective, a much smaller proportion of transmission (minimal in large epidemics) would be from HCWs.

## Discussion

We hypothesize, and illustrate using data, that prophylactic vaccination of HCWs could have a substantial epidemic-reducing effect. Reactive vaccination of HCWs is of limited value in preventing early disease transmission, although reactive vaccination would be an essential humanitarian priority to protect HCWs and maintain the workforce key to controlling any epidemic [9]. Ring-vaccination of cases may well be effective, but even if available, may well not be deployed early in new epidemics and certainly not until the epidemic is recognised and hence potentially already spreading. A prophylactic vaccination campaign, with a vaccine providing long-lasting immunity for all front-line HCWs, either in Ebola epidemic risk areas or where Ebola is present in animal reservoirs, should be seriously considered. It is likely to have a profound impact on the prevention of future outbreaks and epidemics. In some cases, it might stop them completely since, during the early phases of an epidemic (as opposed to later once the epidemic has been recognised), a very large proportion of transmission is via HCWs. Once an epidemic becomes established, the proportion of transmission via HCWs decreases rapidly.

In practical terms, >99 % vaccination coverage equates to approximately 7,400 HCWs (physicians, nurses, and midwives) across Guinea, Liberia, and Sierra Leone, in a population of nearly 22 million [45]. The numbers would be much higher for the wider potentially endemic region. Although it is not a light undertaking, even if a vaccine were available, the huge impact that the recent epidemic had on the lives, healthcare systems, and economies of affected countries, coupled with the risk to the wider world, makes this a worthwhile proposition. A prophylactic vaccination campaign for HCWs, as opposed to the general population, would be easier to administer, more likely to be acceptable, and have higher uptake rates. An example of such a strategy is Hepatitis B vaccination of HCWs in many countries. Whether it could be cost-effective will depend on vaccine efficacy, duration of protection, and pricing structure. However, as these parameters are not currently available, any modelling of this would have little practical use. Nonetheless, based on the illustrative data, investing in developing vaccines with the relevant characteristics (longevity of protection and good side-effect profiles), even if multi-dose, seems a reasonable decision.

Many of the historical outbreaks have been so rural that formal HCWs were not involved in the initial chain of transmission; instead transmission may be via traditional healers and community workers who play a substantial role in healthcare provision and community structure. We have been unable reliably to assess the role of traditional healers in epidemic transmissions due to the paucity of reliable data. However, there is an argument for further investigation of targeted vaccination campaigns of these providers, particularly in known ‘at risk’ areas or reactively after the onset of an outbreak in a nearby area. For example, in Sierra Leone, traditional healers play an integral role in healthcare and community structure and, based on the limited data available [29–31], appear to have acted as early ‘super-spreaders’ of infection.

There are inevitably several limitations to the illustrative analysis that backs up this policy paper. The main one is the paucity of open-access data and the inevitable incompleteness of epidemic trees. The analysis is, therefore, based on a small number of outbreaks with limited information. Furthermore, reported transmission events are biased towards larger events and hospital-based events, meaning HCW association may be over-represented. Finally, the definition of HCWs varies and there is a lack of data regarding the number of HCWs and the specific capacity in which they were working; this makes a denominator difficult to estimate.

In the analysis we assume that outbreaks originate from one single introduction of the virus in to the human population. We believe this to be true in most, but not all, epidemics. Furthermore, we assume there is no significant transmission from asymptomatic individuals, an area of debate in the literature [46]. The assumption of 100 % efficacy for vaccines is, of course, not meant as a formal model of actual impact as the data are not there to support this. Rather it is to illustrate that prophylactic vaccination, if effective, could have effects very different to reactive vaccination of HCWs. A less efficacious vaccine at a higher than 75 % coverage would also have significant effect (data not shown).

This study is intended neither as a fully detailed policy review of all vaccination strategies, nor as a model-based evaluation of different vaccination strategies, but rather to address a single question: should prophylactic vaccination of HCWs, which would require a long-lasting and relatively safe vaccine (but would not require a single-shot or rapidly acting vaccine), be considered as part of the policy process and desirable product profile for Ebola vaccines?

## Conclusion

Serious consideration of a prophylactic HCW Ebola vaccination strategy is needed in countries at threat from Ebola epidemics if a long-protecting vaccine can be developed. The value of vaccinating HCWs is often seen solely in terms of personal protection and maintenance of the health (and morale) of HCWs, where a reactive vaccination strategy would be sufficient if a fast-acting vaccine is available. However, our analysis of the (limited) available empirical data suggests that a prophylactic strategy could bring substantial additional benefits by preventing chains of early transmission before the risk of epidemic spread is recognised.

## Additional file

Additional file 1:
**Transmission trees used in illustrative analysis.** (DOC 169 kb)

## Footnotes

1 The definition of a HCW varies considerably. In this review, we define HCWs as ‘all people engaged in the promotion, protection, or improvement of the health of the population’. Healthcare management and hospital support staff are not included as they do not routinely have direct clinical contact with patients of the sort which tends to increase risk of acquiring Ebola.
