# Supplementary material for: Role of healthcare workers in early epidemic spread of Ebola: policy implications of prophylactic compared to reactive vaccination policy in outbreak prevention and control
Source: BMC Med. 2015 Oct 19;13:271. doi: 10.1186/s12916-015-0477-2 (PMC4612417; doi:10.1186/s12916-015-0477-2)

**SUPPLEMENTARY INFORMATION**

**Additional transmission trees used in illustrative analysis**


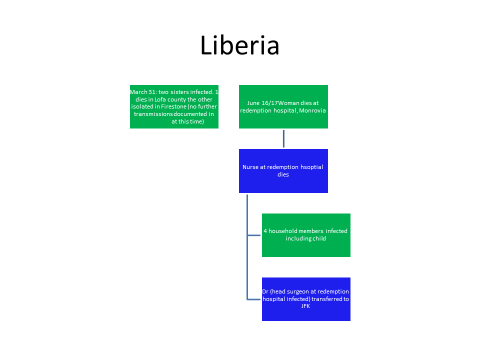


Key:

HCWs

Non-HCWs

Unknown


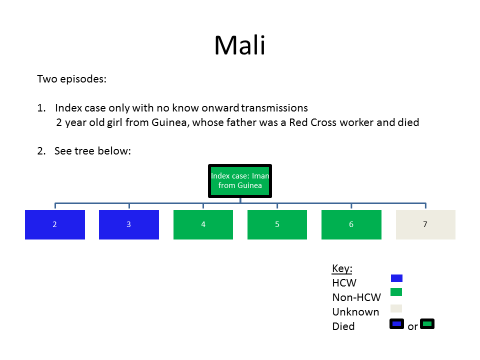


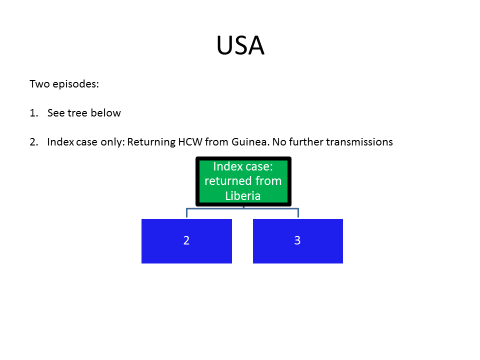


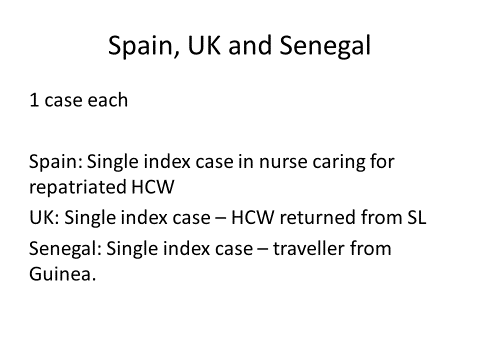


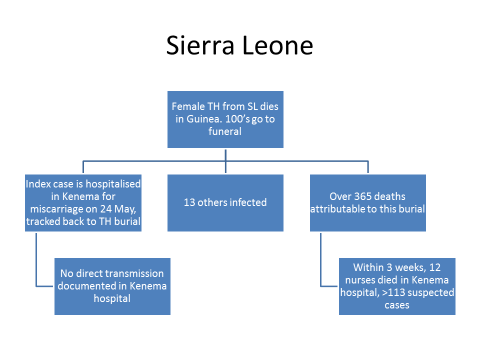

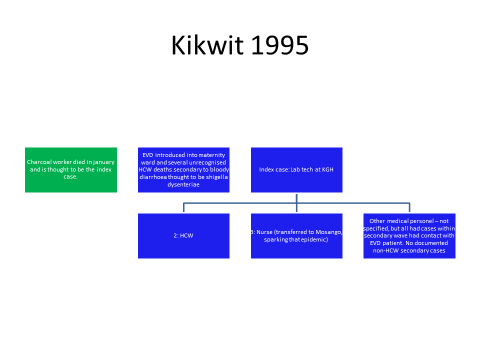

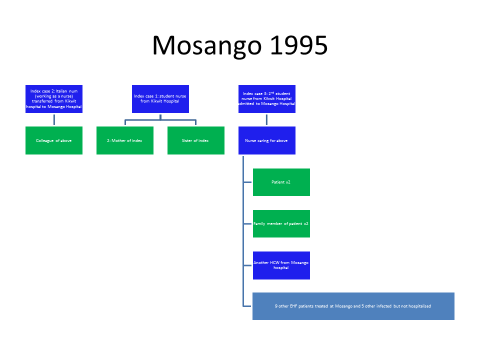

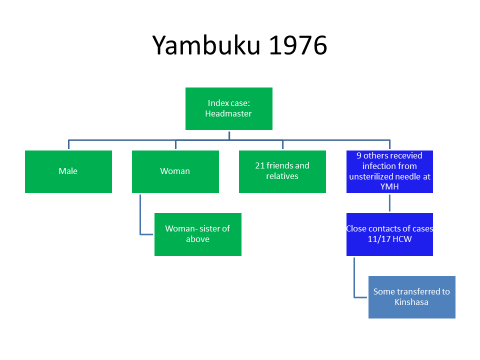

Supplement: Additional file 1: — Transmission trees used in illustrative analysis. (DOC 169 kb) [file 12916_2015_477_MOESM1_ESM.doc]
